# The lifetime of a linac monitor unit ion chamber

**DOI:** 10.1002/acm2.13463

**Published:** 2021-11-11

**Authors:** Ashley J. Cetnar, Dominic J. DiCostanzo

**Affiliations:** ^1^ Department of Radiation Oncology The Ohio State University Columbus Ohio USA

**Keywords:** ion chamber, monitor unit chamber

## Abstract

This study is the first to report the clinical lifetime of Varian Kapton sealed ion chambers as a retrospective review. The data have been analyzed using ion chamber gain values, daily quality assurance results, monthly quality assurance results, and delivered treatment field data were analyzed to comprehensively review trends. The data show the average lifetimes of the ion chambers from our institution, so other physicists can prepare for replacement. Additionally, we share our experience in performing quality assurance tests to calibrate and validate the radiation beam after ion chamber replacement.

## 1 INTRODUCTION

The most common radiation therapy in the United States involves external beam radiation therapy with the use of a linear accelerator (linac).^1^, ^2^ While linacs are now considered commonplace in modern therapy, the machines are complex requiring advanced technical expertise for proper maintenance. One of the critical components to help ensure accurate treatment delivery is the monitor ionization chamber located in the head of the linac. 

The monitor ionization chamber (referred to as monitor chamber) is used for calibrating the linac's absolute dose output and for monitoring the stability of the radiation beam during treatment. The reading of the monitor chamber in monitor units (MU) is related to measured dose under reference conditions using standardized protocols for clinical reference dosimetry. Current recommendations include reports such as the American Association of Physicists in Medicine (AAPM) Task Group (TG) Report 51 or the International Atomic Energy Agency Technical Report Series 398.^3^, ^4^ The monitor chamber is part of a feedback system allowing the linac to automatically control multiple aspects of the radiation beam. While each linac vendor utilizes a unique design for the monitor chamber, Varian (Varian Medical Systems, Palo Alto, CA) currently uses the Kapton sealed ion chamber for the TrueBeam linacs.^5^

Monitor chambers are subject to large quantities of high‐energy radiation, which can cause the sensitivity to drift over time due to oxygen depletion within the chamber.^5^ These changes have been well documented in the literature showing monthly outputs trending upward over time, with the greatest rate of change recorded within the first several months of commissioning.^6^, ^7^, ^8^, ^9^, ^10^ While most of the described data are for early generation linacs or has been limited to single‐institution reports,^6^, ^7^, ^8^, ^9^, ^10^ Bolt et al.^11^ have shown a mean trend for output adjustments of 1.22% per year with trend standard deviation of 2.27% per year when reviewing 96 Varian linacs in a multi‐institutional study from the UK.

While the initial change in sensitivity of the monitor chamber has been well characterized in the literature up to about the first 4 million MU, there currently is no published data available on the lifetime of the monitor chamber. Past publications have noted monitor chamber replacements when reporting output trends,^9^, ^12^ and it has been reported that linac output could be correlated with changes in temperature and pressure due to compromise of the sealed monitor chamber prior to monitor chamber replacement.^13^ This work shares our clinical experience based on the lifetime of Varian Kapton sealed ion chambers as a retrospective review. Data are provided to show the average lifetimes of the monitor chambers and our experience with quality assurance tests performed after monitor chamber replacement, so other physicists can be better prepared for possible failures (defined as necessity for replacement). In addition to the output trends, this is the first known study to include gain values for reporting monitor chamber response utilizing a physical quantity of the chamber itself.

## 2 MATERIALS AND METHODS

**FIGURE 1:**
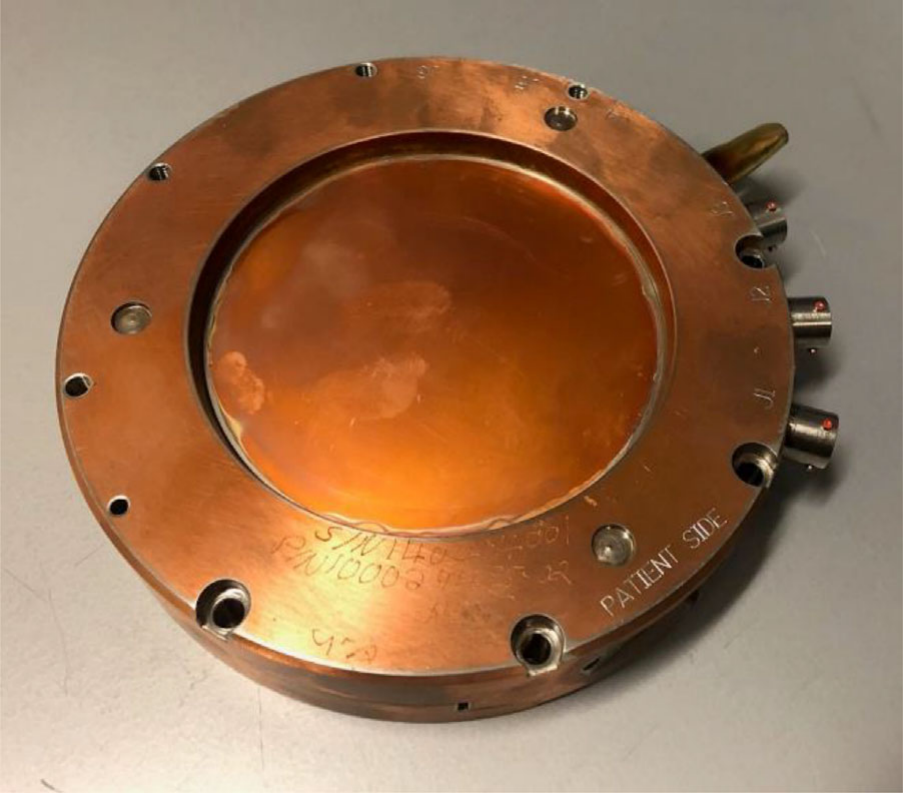
Kapton monitor chamber (P/N100029495‐02) used in Varian TrueBeam

Varian uses a Kapton sealed ion c hamber (P/N100029495‐02) for TrueBeam linacs made of two independently sealed volumes with windows and electrodes made of radiation‐resistant polyamide resin called Kapton shown in Figure 1.^5^ Mechanical and chemical properties of Kapton have been characterized in high radiation environments.^14^ The monitor chamber is designed with primary and secondary chambers for redundancy organized in segments to also detect changes in flatness and symmetry.^14^

The beam symmetry is monitored with an interlock system preventing beam delivery if deviations exceed the set threshold based on the sectors of the monitor chamber. Prior to reaching the threshold, the monitor chamber relays measured beam intensity from four quadrants to the steering coils allowing for minor adjustments of the beam to ensure symmetry. Additionally, the dose rate is monitored allowing the linac to increase or decrease the dose rate to maintain the stability of the beam. The most common indication of monitor chamber failure is when faults 212022 (Flatness Radial) and 212023 (Flatness Transverse) are frequent. These faults are triggered due to differences in monitor unit chamber flatness readings exceeding 10% and is a typical symptom of the end of life for the chamber.

Our institution is responsible for nine (*N* = 9) clinical Varian linacs including the TrueBeam, TrueBeam STx, and Edge models shown in Table 1. Linacs were commissioned from 2011 to 2014 with six commissioned in 2014 corresponding to the opening of a new cancer hospital. The machines used in this study are all clinical linacs that treat a variety of disease sites. However, each machine in our institution has a primary disease site. The linacs of types Edge or TrueBeam STx (linacs in vaults 2, 6, and 7) treat patients with head‐and‐neck cancer, primary brain cancer, and a significant portion of our stereotactic (both cranial and extracranial) patient volume. In contrast, linac in vault 8 is used for special procedures including total body irradiation and total skin irradiation, while linacs in vaults 1 and 3 treat the majority of the breast cancer patients. The remaining machines treat an amalgamation of other disease sites including cancers of the thorax, abdomen, and prostate. Data reported include the linac model, dates of acceptance, dates of monitor chamber replacement, and the clinically delivered MU at the time of replacement.

**TABLE 1:** Summary of data for clinical linacs

**Chronological linac number**	**Linac type**	**Primary disease sites treated**	**Acceptance date**	**Ion chamber replacement**	**Clinically delivered MU**
1	Varian TrueBeam	Breast	4/28/2011	11/30/2018	19 049 770
2	Varian STx	Head and neck, brain, and stereotactic	2/1/2012	11/6/2015	16 164 602
2	Varian STx	Head and neck, brain, and stereotactic		5/28/2019	14 215 864
3	Varian TrueBeam	Breast	9/15/2013	10/9/2019	15 049 344
4	Varian TrueBeam	Various	6/24/2014	3/7/2019	14 798 379
5	Varian TrueBeam	Various	6/24/2014	9/13/2018	12 963 455
5	Varian TrueBeam	Various		1/31/2020	5 456 543
6	Varian Edge	Head and neck, brain, and stereotactic	8/14/2014	8/9/2019	17 381 866
7	Varian Edge	Head and neck, brain, and stereotactic	9/8/2014	10/29/2018	18 127 792
8	Varian TrueBeam	Special procedures	9/24/2014	9/6/2018	13 681 860
9	Varian TrueBeam	Various	9/30/2014	2/21/2017	7 700 326

The monitor chamber gain data were acquired from the eXtensible Markup Language (XML) files saved by the console. After a change is made modifying stored parameters used by the linac in service mode, the user is required to actively save the changes. Upon storing of the parameters to the linac, an XML file is written to a mirrored directory for logging change history. Custom Python software was written to search the directories where the XML files are stored, read the appropriate saved configurations, and store the data alongside the clinical output data.

The data for the daily output was acquired with the Daily QA 3 (SunNuclear Melbourne, FL) prior to the start of each treatment day. The monthly output data were acquired using a Farmer‐type ionization chamber in GAMMEX Solid Water during the performance of routine QA. Finally, the total MU delivered during treatment was estimated by querying our record and the system was verified by analyzing the MU for treatment fields delivered by each linac. 

We investigated the length of time before monitor chamber replacement, beam hours, and filament hours from routine preventative maintenance inspections (PMI). Since PMIs were performed quarterly, the reported values for beam and filament hours are reported for the closest date to the monitor chamber replacement. The estimated clinical MU delivered, cumulative MU at monitor chamber replacement, and elapsed time before replacement is summarized. For this study, all aforementioned data for all clinical linacs were exported and analyzed using a combination of Microsoft Excel and custom Python programs. 

## 3 RESULTS

Table 1 provides a summary of the clinical data for our Varian linacs in our clinic including the linac model, acceptance date, initial date of monitor chamber replacement, and the total number of MU delivered at the time of replacement.** ** If a linac had an additional monitor chamber replacement, the second date are listed with the clinically delivered MU during the lifetime of the second chamber.

**FIGURE 2:**
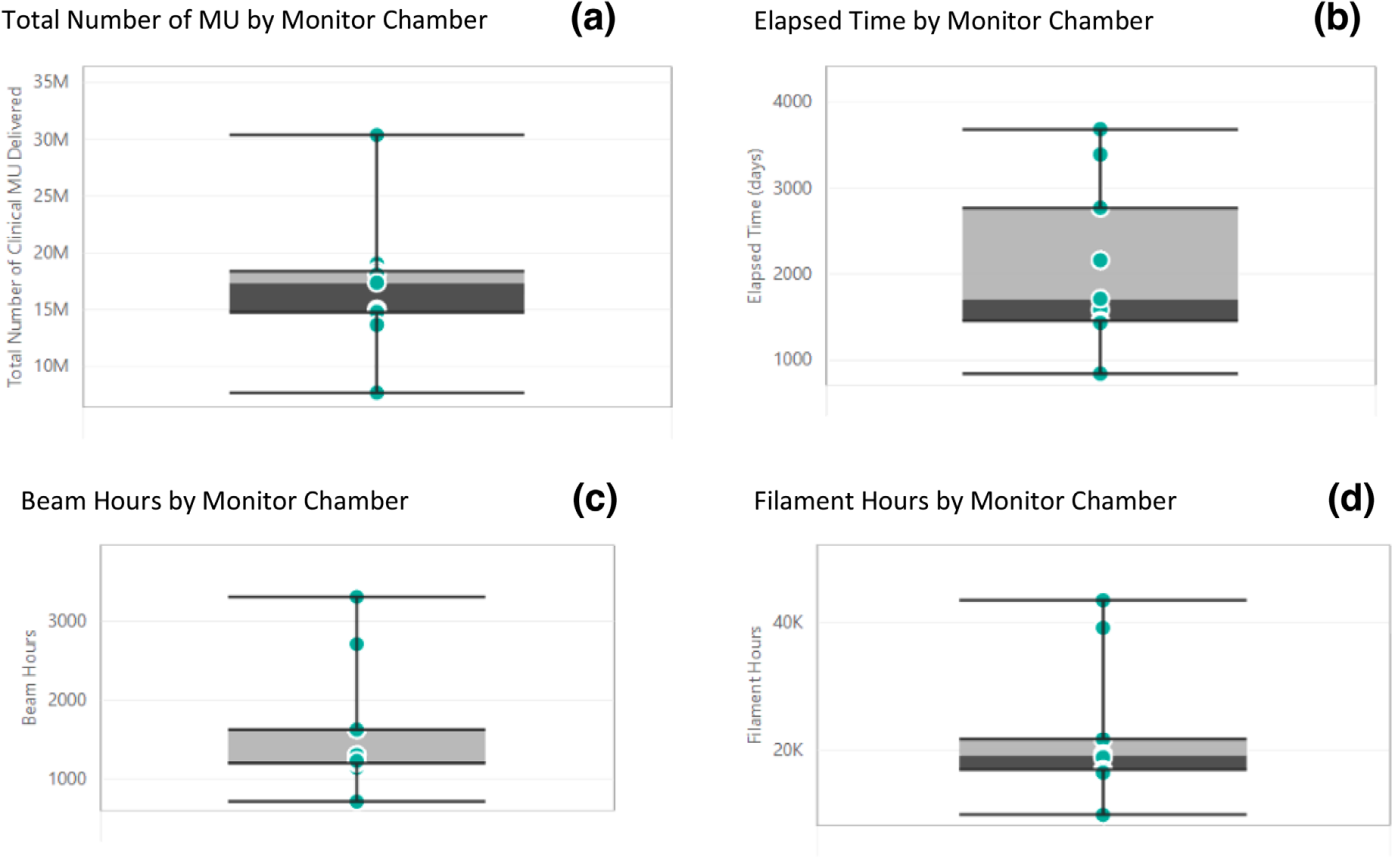
Data for clinical monitor units (MU) delivered (a), elapsed lifetime before replacement (b), number of beam hours (c), and number of filament hours from replaced ion chambers (d) across all monitor chambers

Figure 2 summarizes the estimated total number of clinical MU delivered, elapsed lifetime before replacement, and number of beam and filament hours from the preventative maintenance reports for each ion chamber that has been replaced.  

Table 2 shows the total number of MU delivered on the linac at the time of ion chamber replacement. The average number of MU delivered per machine before ion chamber replacement was 13.9 million (STDEV 4.1 million). The average amount of time before the first ion chamber replacement was 1731 days (STDEV 567), which is approximately 4.75 years for the workload in our clinic.

**TABLE 2:** Monitor unit (MU) delivered, elapsed time, beam hours, and filament hours on the linac at the time of ion chamber replacement

	**Total number of clinical MU delivered**	**Elapsed time (days)**	**Beam hours**	**Filament hours**
Average	13 937 821	1731	1312	18 640
Standard deviation	4 144 750	569	317	4418

**FIGURE 3:**
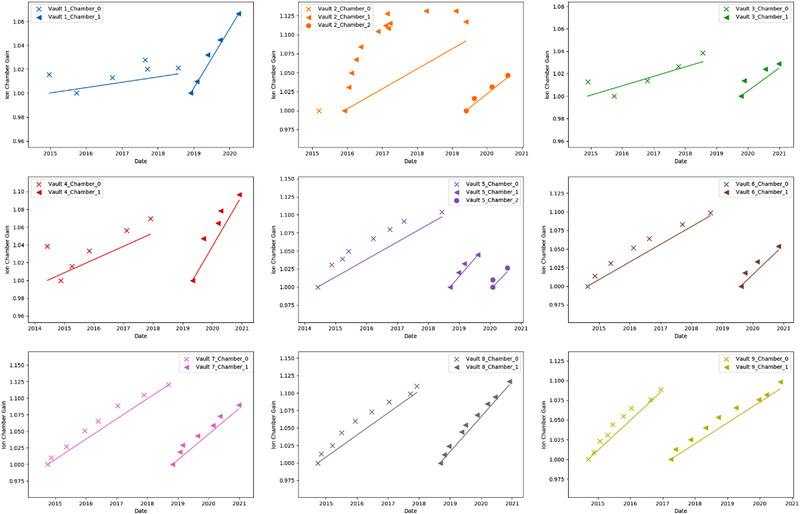
Changes in monitor chamber gain for each linac over time

Figure 3 shows changes to gain for each monitor chamber over time including the original and replaced chambers since there has been the ability to write values to XML files from the console software. The gain values are normalized to first recorded value, and when multiple adjustments were made at the time of acceptance, the first value displayed for the monitor chamber is not equal to one. A linear trend line is displayed for each chamber to represent output adjustment made over time with the original ion chamber denoted as Chamber 0 and subsequent replacement chambers as Chambers 1 and 2. In most cases, the data show incremental changes in output over time without a clear plateau. There is an outlier for linac 2's second monitor chamber, showing a rapid increase in uncorrected output before plateauing. However, the output varied with a large standard deviation, and this chamber was replaced due to significant variation of output on a daily basis requiring frequent output adjustments (daily or every other day).

**FIGURE 4:**
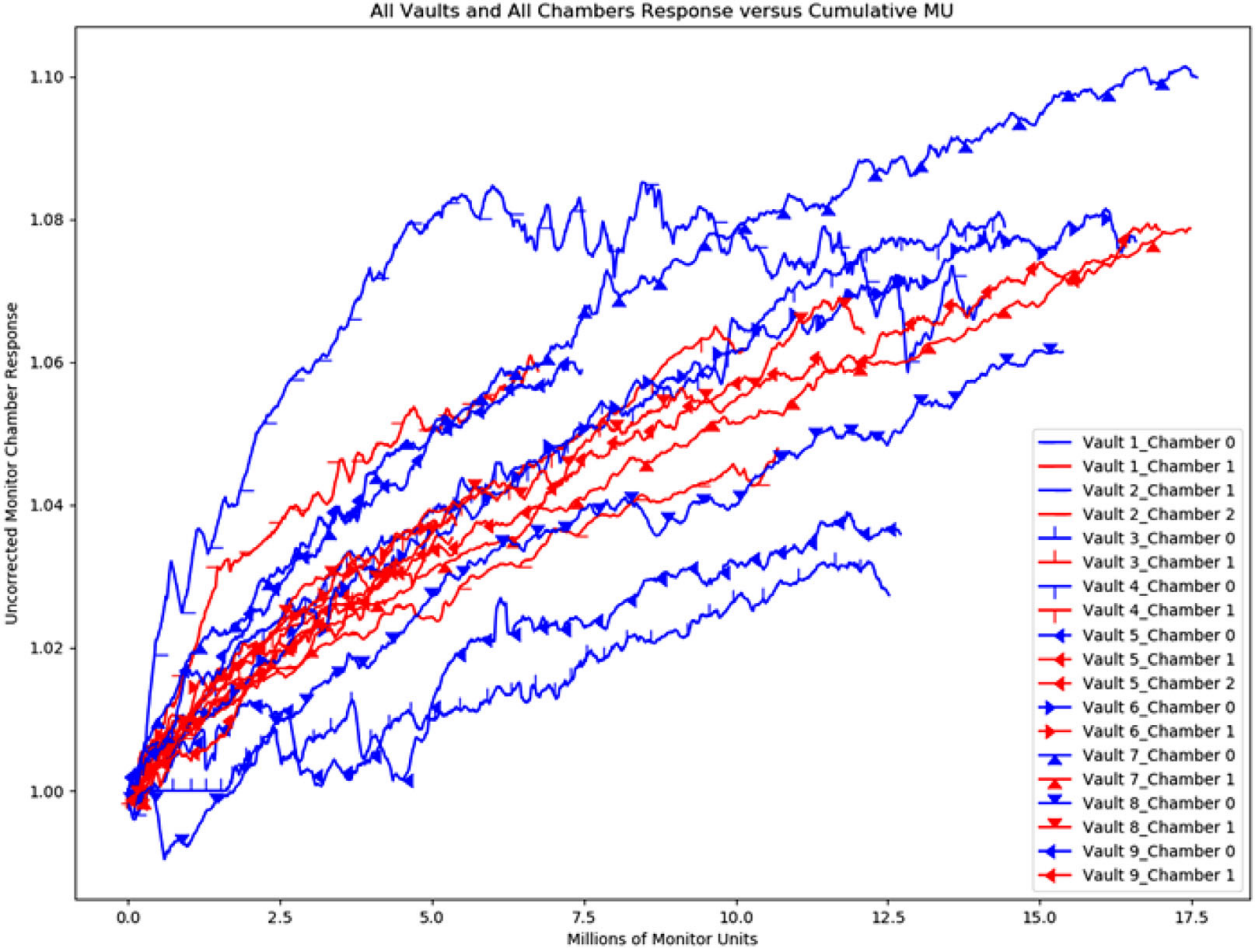
Changes in daily linac output by clinical monitor units delivered per monitor chamber based on uncorrected monitor chamber response reported as a moving average of 20 data points (approximately one working month)

As seen in Figure 4, the monitor chambers follow a similar trend as described in the literature with output drifting upward based on data collected from the daily Quality Assurance (QA) output measurements, but our data do not support that these output changes stabilize over time. Data based on uncorrected monitor chamber response are displayed as a moving average of 20 data points (approximately one working month) with blue trend lines representing the original monitor chamber from linac acceptance (chamber 0) and red trend lines representing subsequent replacement chambers (Chambers 1 and 2).  The missing Daily QA data for Vault 3 Chamber 0 is represented by horizontal line as a result of an upgrade in software. Data for each linac can be reviewed in the Supporting Information.

**FIGURE 5:**
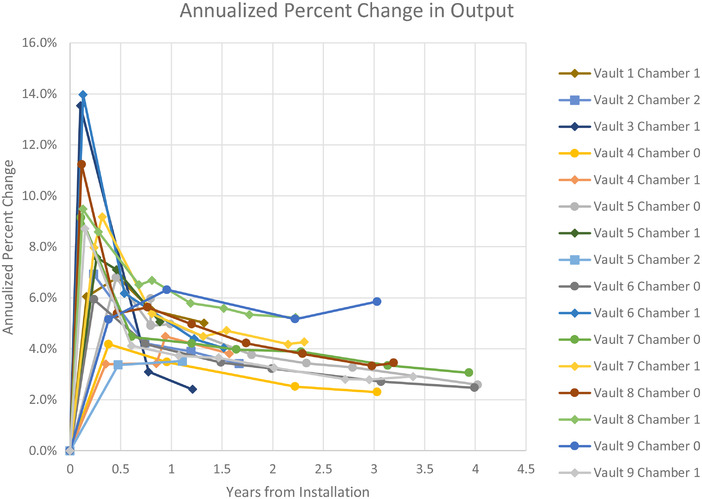
Annualized percent change in output per monitor chamber where Chamber 0 represents the initial chamber from acceptance and calibration, with Chambers 1 and 2 representing subsequent replacement monitor chambers per linear accelerator by vault

The change in output can also be considered by the cumulative output adjustments over the course of a year. Figure 5 shows large changes in output over the first 6 months of a monitor chamber lifetime. While the changes in output are shown to be predictable over the lifetime of the chamber, our experience has shown required output adjustments ranging between 2% and 6% per year. To aid in the visualization of the required changes to output in shorter periods, each adjustment to the output was divided by the time, by days, since the chamber was installed. For instance, if the output was adjusted by 3% after 182 days, the annualized output change would be calculated as:

$$6.0\%\;=\;3\%*\;\left(\frac{365\;\text{days}}{182\;\text{days}}\right)$$

## 4 DISCUSSION

The trends would suggest that when a monitor chamber measures approximately 14 million clinically delivered MU, physicists should be prepared for an increased likelihood of replacement. However, it may not be trivial for readers to estimate and track the number of MU delivered in the clinic, so the results for other quantitative surrogates including elapsed time, beam hours, and filament hours per linac are also reported in Table 2 and Figure 2 for reference. Trending daily or monthly output data may provide an early indication of failure due to the plateau or stabilization of output. 

The data presented represent estimates of clinically delivered MU. MU delivered due to routine quality assurance or other linac service is not presented in the data in this study, but should be considered if there are changes to quality assurance over the lifetime of the linac. We estimate that approximately 750 000 MU are delivered for each of our linacs each year for daily, monthly, and annual QA testing based on AAPM TG‐142.^15^ However, because these procedures have been standardized in our clinic, we expect this value to be relatively constant across our clinical linacs. 

One important difference for a replacement monitor chamber is the stabilization period for output. Typically, during commissioning, a large number of MUs are delivered for quality assurance measurements, which helps to bring the chamber to the plateau earlier during clinical treatments. However, if a monitor chamber is replaced, the linac is typically returned to clinical service soon after the maintenance, so outputs should be closely monitored as the chamber stabilizes over time. While data presented here shows general trends for clinical wear‐and‐tear for the linac, the reason for monitor chamber failure is also of interest. We observe two‐chamber replacements as outliers in our data replaced earlier than the data would suggest a replacement was needed. After reviewing the service reports for replacement, one was replaced due to intermittent flatness faults, but specific details were not noted in the report for the other early replacement.

Interestingly, one monitor chamber in our clinic suffered a unique failure mode by which both sides of the chamber deflated simultaneously. After investigation by the manufacturer, it was found that the leak was through both filler tube pinch‐offs, not through the chamber body. An improvement to the manufacturing process was implemented in 2018 to mitigate this failure mode for newer monitor chambers. The manufacturer also suggested that this type of failure mode is more common when the chamber is in a cooled state for more than 24 h, as was the case for this machine which suffered a separate and unrelated failure requiring the machine to sit idle in a powered‐off state (not standby) for more than 24 h. In our experience, a physicist can expect to see early warning signs of faults on the linac that become more frequent as the chamber fails. It is unlikely that the chamber fails suddenly and catastrophically.

In the cases where the chamber fails gradually, preparation for the replacements can be undertaken as summarized in Table 3. In our institution, we have used a diode array that allows for real‐time measurements to capture baseline profiles for each energy. After replacement, these baselines allow for efficient beam steering and validate the beam flatness and symmetry return to the previous clinically acceptable limits. After the steering takes place (if required), the monthly beam profile QA is performed for each energy to ensure a return to baseline within the tolerances set by AAPM TG‐142.^15^ Additional tests that we perform after monitor chamber replacement include light versus X‐ray coincidence for each energy and light field alignment. To verify light field alignment, we verify collimator walkout and symmetry of field sizes. The collimator is set at 0° with a set field size (i.e., 10 cm x 10 cm) projected on a piece of paper and is marked to indicate the field edges. The collimator is rotated to cardinal angles (90° and 270°) and the field size is visualized so that the field edges match the scribed sizes from a start position of a 0° collimator angle.

**TABLE 3:** Example of physics quality assurance for monitor chamber replacement

Pre‐replacement	Obtain baseline profiles for each energy
Post‐replacement	Beam steering and validation of flatness and symmetry based on baseline profiles
	Verification of light field and X‐ray coincidence
	Output verification
	Monthly flatness and symmetry

Following monitor chamber replacement, the absolute output of the linac will often require adjustments that are larger than the adjustments made on a routine basis. In many cases, we were required to make adjustments of output by increasing it nearly 25% after replacement. Unlike the results of Bolt et al.^11^ showing a mean trend of a 1.22 ± 2.27% per year, we are observing much greater changes in output in the first year and trends of cumulative output changes between 2% and 6% throughout the year over the lifetime of the chamber. Since the chambers are pressurized, the magnitude and direction of the adjustment can be described by the physical processes of chamber deflation, which is one of the most common failure modes. When a new chamber is installed, the gain of the chamber defaults to the gain set on the previous chamber. The new pressurized chamber will have more molecules of gas in the chamber.  As a result, when a set amount of radiation is delivered to the new chamber, the chamber will collect more ion pairs per pulse of radiation causing the MU count to increase more quickly than with the old chamber. As a result, the gain must be reduced. In early versions of the console software, large changes in output would pose problems that could unintentionally corrupt the configuration files. As a result, an increased number of smaller adjustments were required. However, in the current version of the software, large adjustments are possible.

While limited predictive power can be attributed to these results, it is possible to identify trends, and with future replacements providing additional data, we expect it may be possible to hone models for prediction based upon the results shown. Data have been provided from nine linacs from a single institution in this review to explore potential relationships and patterns between variables. Additional data could be investigated from anonymized service log/replacement data from Varian or other sites as a multi‐institutional review for a more comprehensive review of the lifetime of a monitor chamber.

## 5 CONCLUSIONS

An institutional review and analysis of monitor chamber replacements has been presented with the average lifetime of a monitor chamber is approximately 14 million clinically delivered MU. We describe the relationship between the uncorrected output and cumulative MU delivered to each chamber and identify and describe outliers within this study. We have found replaced chambers have greater initial changes in output than would be anticipated based on current literature for output changes, and we would recommend this be closely monitored after replacement. We did not observe a plateau in the output changes over time as suggested by other reports. A review of our institutional processes following replacement are described to provide some structure as to what tests are performed and what a physicist can expect upon replacement of a monitor chamber.

## CONFLICT OF INTEREST

The authors declare no conflict of interest.

## AUTHOR CONTRIBUTION STATEMENT

Ashley Cetnar: Conceptualization, Methodology, Investigation, Visualization, and Writing – Original Draft Preparation Dominic DiCostanzo: Methodology, Investigation, Software, Data Curation, Formal Analysis, Visualization, and Writing – Review & Editing.

## Supporting information

Supporting information

## Data Availability

The data that support the findings of this study are available from the corresponding author upon reasonable request.

## References

[1] OECD . Radiotherapy equipment. Accessed 13 April, 2021. https://data.oecd.org/healtheqt/radiotherapy‐equipment.htm

[2] American Cancer Society . Getting external beam radiation therapy. 2019.

[3] Almond PR , Biggs PJ , Coursey BM , et al. AAPM's TG‐51 protocol for clinical reference dosimetry of high‐energy photon and electron beams. Med Phys. 1999;26(9):1847‐1870. 10505874 10.1118/1.598691

[4] Andreao P , Burns DT , Hohlfeld K , et al. IAEA TRS‐398: absorbed dose determination in external beam radiotherapy: an international code of practice for dosimetry based on standards of absorbed dose to water. IAEA; 2006.

[5] Purwar A , Johnse S , Potter R . Performance charactersitics of Kapton sealed ion chambers. Varian Medical Systems, Inc: Palo Alto, CA; 2016.

[6] Glide‐Hurst C , Bellon M , Foster R , et al. Commissioning of the Varian TrueBeam linear accelerator: a multi‐institutional study. Med Phys. 2013;40(3):031719. 23464314 10.1118/1.4790563

[7] Kapanen M , Tenhunen M , Hämäläinen T , Sipilä P , Parkkinen R , Järvinen H . Analysis of quality control data of eight modern radiotherapy linear accelerators: the short‐and long‐term behaviours of the outputs and the reproducibility of quality control measurements. Phys Med Biol. 2006;51(14):3581. 16825750 10.1088/0031-9155/51/14/020

[8] Hossain M . Output trends, characteristics, and measurements of three megavoltage radiotherapy linear accelerators. J Appl Clin Med Phys. 2014;15(4):137‐151. 10.1120/jacmp.v15i4.4783 PMC4393854 25207404

[9] Grattan M , Hounsell A . Analysis of output trends from Varian 2100C/D and 600C/D accelerators. Phys Med Biol. 2010;56(1):N11. 21119232 10.1088/0031-9155/56/1/N02

[10] Binny D , Lancaster CM , Kairn T , Trapp JV , Crowe SB . Monitoring Daily QA 3 constancy for routine quality assurance on linear accelerators. Physica Med. 2016;32(11):1479‐1487. 10.1016/j.ejmp.2016.10.021 27839928

[11] Bolt MA , Clark CH , Chen T , Nisbet A . A multi‐centre analysis of radiotherapy beam output measurement. Phys Imaging Radiat Oncol. 2017;4:39‐43.

[12] Binny D , Aland T , Archibald‐Heeren BR , Trapp JV , Kairn T , Crowe SB . A multi‐institutional evaluation of machine performance check system on treatment beam output and symmetry using statistical process control. J Appl Clin Med Phys. 2019;20(3):71‐80. 10.1002/acm2.12547 PMC6414149 30786139

[13] McCaw TJ , Barraclough BA , Belanger M , Besemer A , Dunkerley DA , Labby ZE . Diagnosing atmospheric communication of a sealed monitor chamber. J Appl Clin Med Phys. 2020;21(8):309‐314. 32648368 10.1002/acm2.12975 PMC7484838

[14] Megusar J . Low temperature fast‐neutron and gamma irradiation of Kapton® polyimide films. J Nucl Mater. 1997;245(2‐3):185‐190.

[15] Klein EE , Hanley J , Bayouth J , et al. Task Group 142 report: quality assurance of medical acceleratorsa. Med Phys. 2009;36(9Part1):4197‐4212. 19810494 10.1118/1.3190392

