# Supplementary material for: The lifetime of a linac monitor unit ion chamber
Source: J Appl Clin Med Phys. 2021 Nov 11;22(12):108–14. doi: 10.1002/acm2.13463 (PMC8664141; doi:10.1002/acm2.13463)
Supplement: Supplementary file 1 — Supporting information [file ACM2-22-108-s001.docx]

# **Supplementary Material**

Chamber response by linac based on daily QA output are shown in the following figures. The different colors in each region represent daily QA output results between periods of output changes made by the physicist when necessary during monthly or annual QA.

There is an outlier for linac 2’s second ion chamber, which shows a rapid increase in uncorrected output for the first five-million MU before displaying a stabilization in Fig. A1. During this period of stabilization, the output varied with a larger standard deviation than the output exhibited during the upward trend. As mentioned previously, this chamber was replaced due to significant variation of output on a daily basis, which required output adjustments daily or every two days.

The other outlier can be seen in Fig. A5 for linac 5 where there is oscillation between increasing and decreasing chamber response during the first five-million MU. After which this chamber exhibits the expected upward trend before requiring replacement at nearly 12.5 million MU. Upon investigation, there was no evidence of device change, output changes, or other modifications that would explain this behavior.


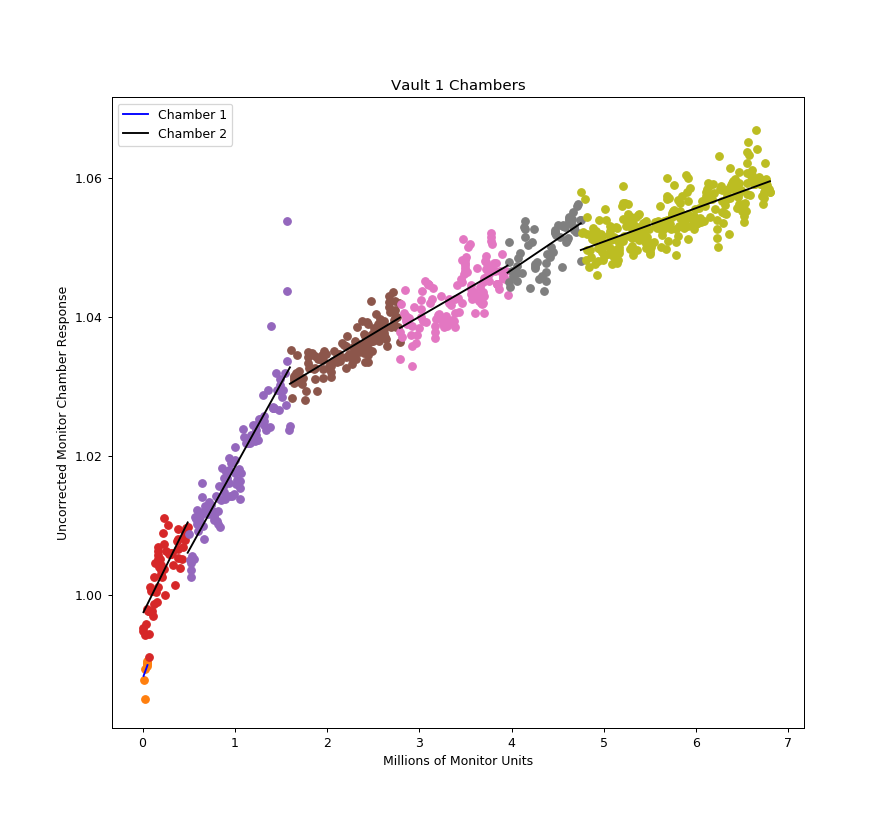


Figure A1: Chamber response based on daily QA measurements for Linac 1


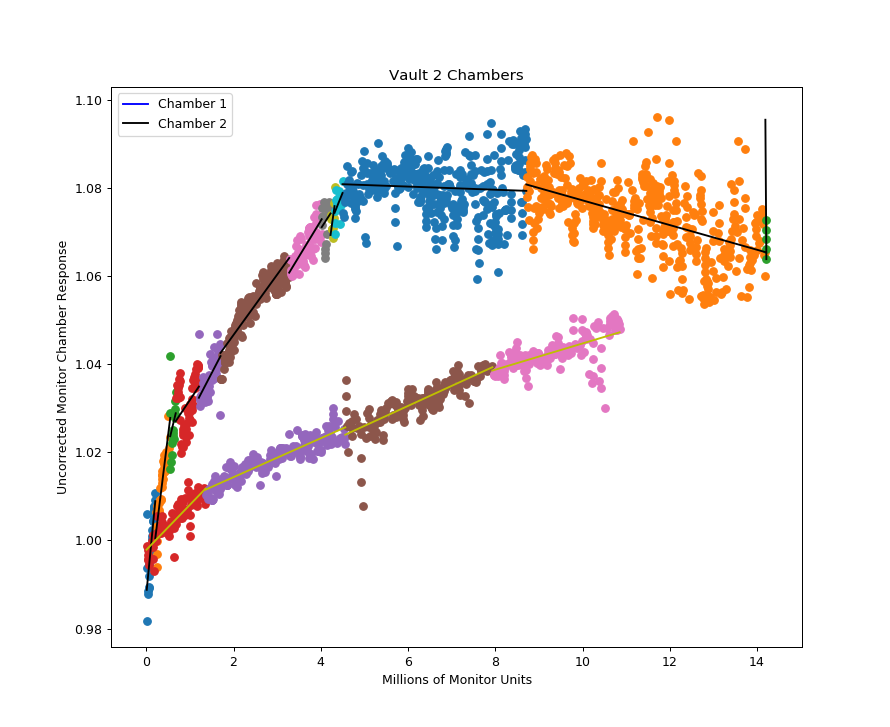


Figure A2: Chamber response based on daily QA measurements for Linac 2


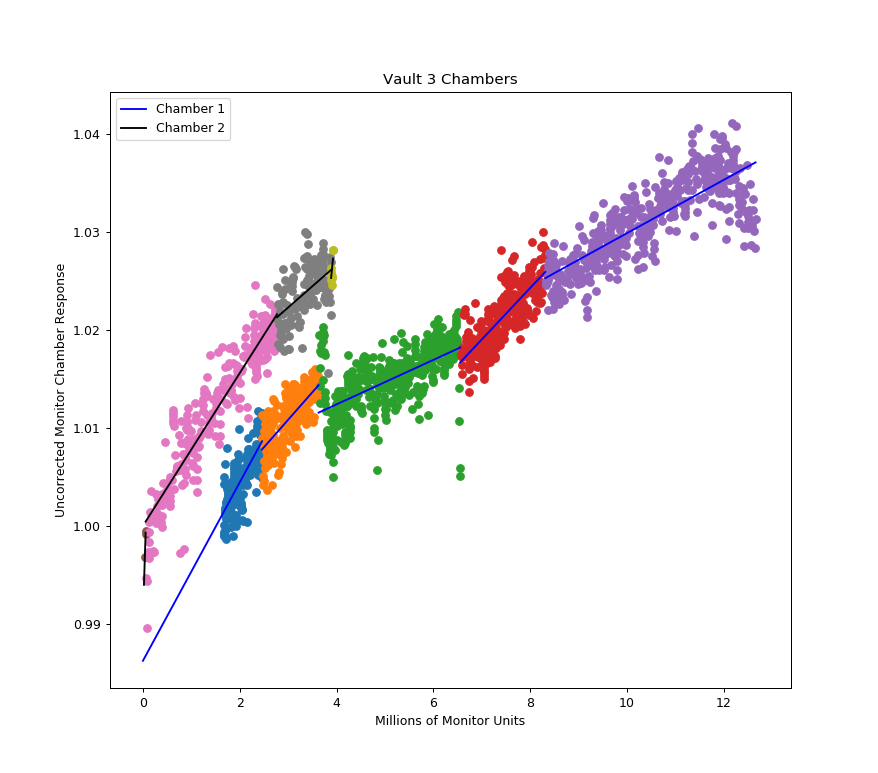


Figure A3: Chamber response based on daily QA measurements for Linac 3


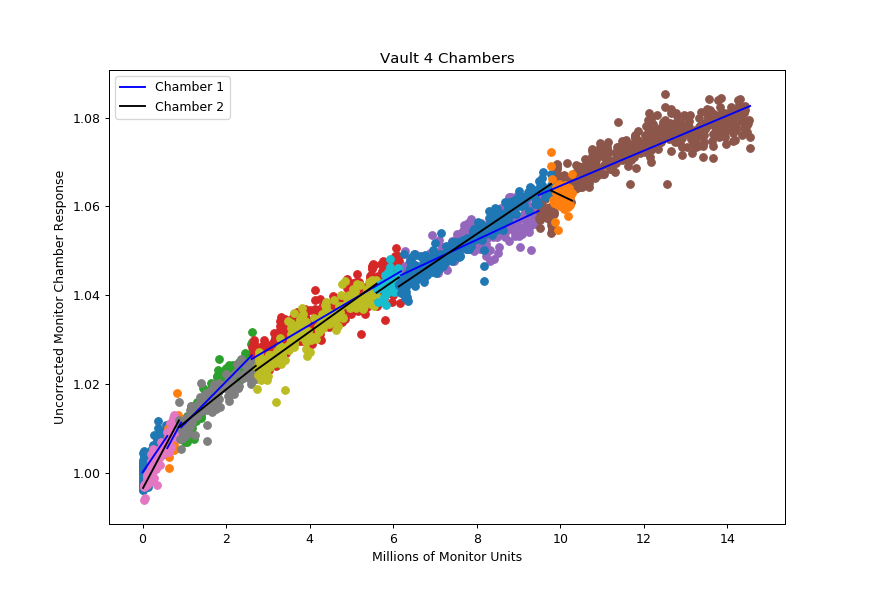


 Figure A4: Chamber response based on daily QA measurements for Linac 4


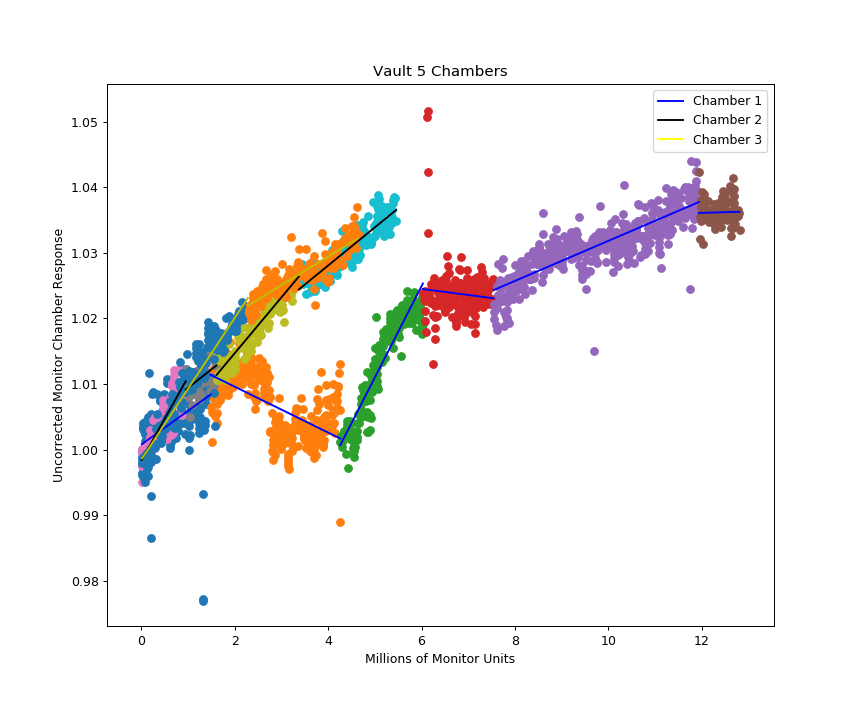


Figure A5: Chamber response based on daily QA measurements for Linac 5


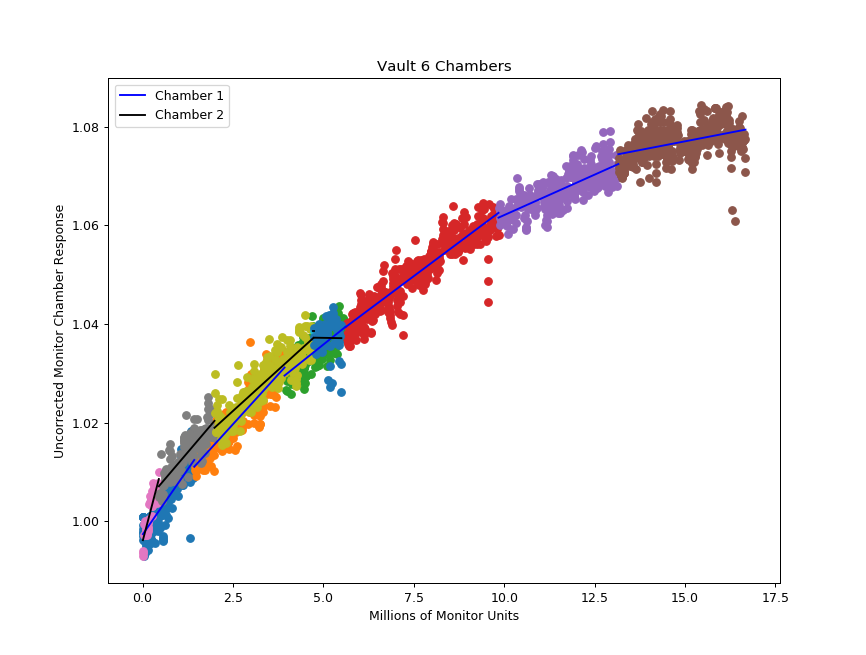


Figure A6: Chamber response based on daily QA measurements for Linac 6


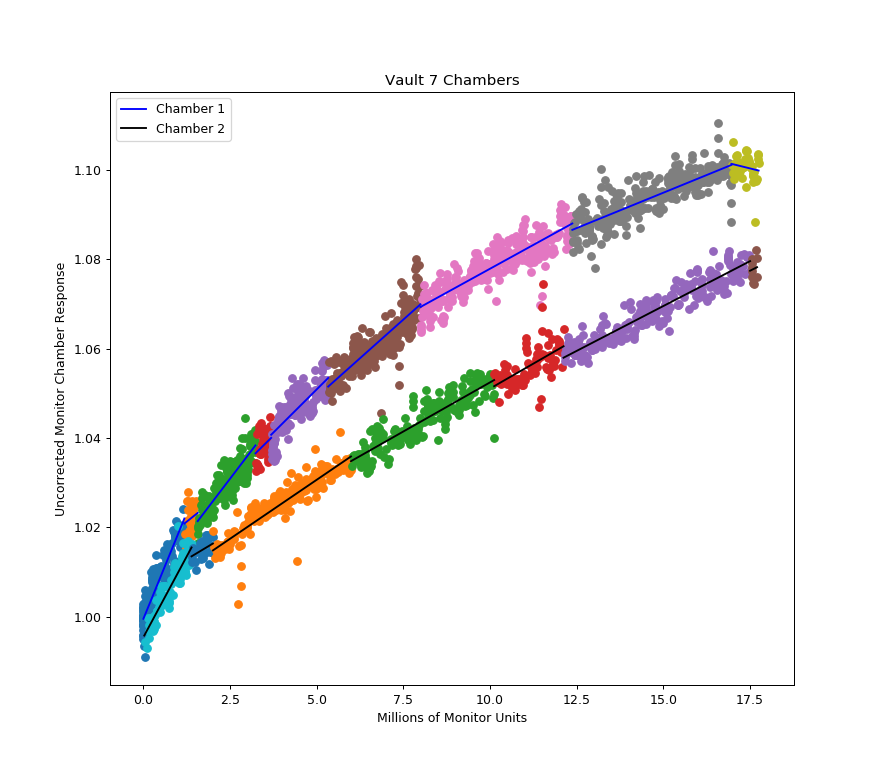


Figure A7: Chamber response based on daily QA measurements for Linac 7


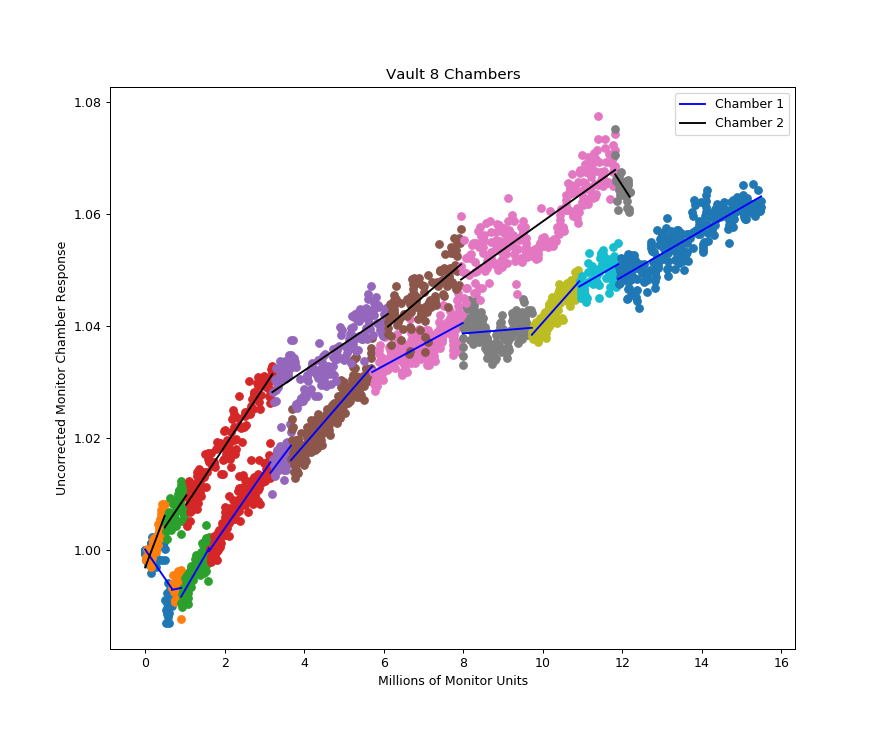


Figure A8: Chamber response based on daily QA measurements for Linac 8


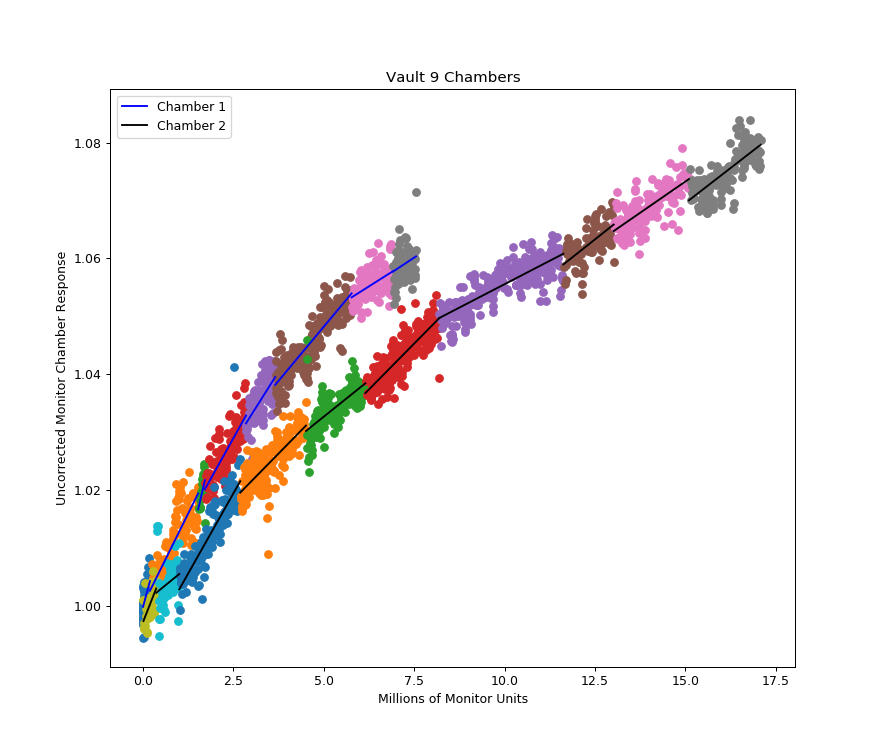


Figure A9: Chamber response based on daily QA measurements for Linac 9
